# Staging tau pathology with tau PET in Alzheimer’s disease: a longitudinal study

**DOI:** 10.1038/s41398-021-01602-5

**Published:** 2021-09-18

**Authors:** Shi-Dong Chen, Jia-Ying Lu, Hong-Qi Li, Yu-Xiang Yang, Jie-Hui Jiang, Mei Cui, Chuan-Tao Zuo, Lan Tan, Qiang Dong, Jin-Tai Yu, for the Alzheimer’s Disease Neuroimaging Initiative, Michael W. Weiner, Paul Aisen, Ronald Petersen, Clifford R. Jack, William Jagust, John Q. Trojanowki, Arthur W. Toga, Laurel Beckett, Robert C. Green, Andrew J. Saykin, John C. Morris, Richard J. Perrin, Leslie M. Shaw, Maria Carrillo, William Potter, Lisa Barnes, Marie Bernard, Hector González, Carole Ho, John K. Hsiao, Jonathan Jackson, Eliezer Masliah, Donna Masterman, Ozioma Okonkwo, Richard Perrin, Laurie Ryan, Nina Silverberg, Adam Fleisher, Diana Truran Sacrey, Juliet Fockler, Cat Conti, Dallas Veitch, John Neuhaus, Chengshi Jin, Rachel Nosheny, Miriam Ashford, Derek Flenniken, Adrienne Kormos, Michael Rafii, Rema Raman, Gustavo Jimenez, Michael Donohue, Devon Gessert, Jennifer Salazar, Caileigh Zimmerman, Yuliana Cabrera, Sarah Walter, Garrett Miller, Godfrey Coker, Taylor Clanton, Lindsey Hergesheimer, Stephanie Smith, Olusegun Adegoke, Payam Mahboubi, Shelley Moore, Jeremy Pizzola, Elizabeth Shaffer, Brittany Sloan, Danielle Harvey, Arvin Forghanian-Arani, Bret Borowski, Chad Ward, Christopher Schwarz, David Jones, Jeff Gunter, Kejal Kantarci, Matthew Senjem, Prashanthi Vemuri, Robert Reid, Nick C. Fox, Ian Malone, Paul Thompson, Sophia I. Thomopoulos, Talia M. Nir, Neda Jahanshad, Charles DeCarli, Alexander Knaack, Evan Fletcher, Duygu Tosun-Turgut, Stephanie Rossi Chen, Mark Choe, Karen Crawford, Paul A. Yushkevich, Sandhitsu Das, Robert A. Koeppe, Eric M. Reiman, Kewei Chen, Chet Mathis, Susan Landau, Nigel J. Cairns, Erin Householder, Erin Franklin, Haley Bernhardt, Lisa Taylor-Reinwald, Leslie M. Shaw, John Q. Trojanowki, Magdalena Korecka, Michal Figurski, Karen Crawford, Scott Neu, Andrew J. Saykin, Kwangsik Nho, Shannon L. Risacher, Liana G. Apostolova, Li Shen, Tatiana M. Foroud, Kelly Nudelman, Kelley Faber, Kristi Wilmes, Leon Thal, Zaven Khachaturian, John K. Hsiao, Lisa C. Silbert, Betty Lind, Rachel Crissey, Jeffrey A. Kaye, Raina Carter, Sara Dolen, Joseph Quinn, Lon S. Schneider, Sonia Pawluczyk, Mauricio Becerra, Liberty Teodoro, Karen Dagerman, Bryan M. Spann, James Brewer, Helen Vanderswag, Adam Fleisher, Jaimie Ziolkowski, Judith L. Heidebrink, Lisa Zbizek-Nulph, Joanne L. Lord, Sara S. Mason, Colleen S. Albers, David Knopman, Kris Johnson, Javier Villanueva-Meyer, Valory Pavlik, Nathaniel Pacini, Ashley Lamb, Joseph S. Kass, Rachelle S. Doody, Victoria Shibley, Munir Chowdhury, Susan Rountree, Mimi Dang, Yaakov Stern, Lawrence S. Honig, Akiva Mintz, Beau Ances, David Winkfield, Maria Carroll, Georgia Stobbs-Cucchi, Angela Oliver, Mary L. Creech, Mark A. Mintun, Stacy Schneider, David Geldmacher, Marissa Natelson Love, Randall Griffith, David Clark, John Brockington, Daniel Marson, Hillel Grossman, Martin A. Goldstein, Jonathan Greenberg, Effie Mitsis, Raj C. Shah, Melissa Lamar, Patricia Samuels, Ranjan Duara, Maria T. Greig-Custo, Rosemarie Rodriguez, Marilyn Albert, Chiadi Onyike, Leonie Farrington, Scott Rudow, Rottislav Brichko, Stephanie Kielb, Amanda Smith, Balebail Ashok Raj, Kristin Fargher, Martin Sadowski, Thomas Wisniewski, Melanie Shulman, Arline Faustin, Julia Rao, Karen M. Castro, Anaztasia Ulysse, Shannon Chen, Mohammed O. Sheikh, Jamika Singleton-Garvin, P. Murali Doraiswamy, Jeffrey R. Petrella, Olga James, Terence Z. Wong, Salvador Borges-Neto, Jason H. Karlawish, David A. Wolk, Sanjeev Vaishnavi, Christopher M. Clark, Steven E. Arnold, Charles D. Smith, Gregory A. Jicha, Riham El Khouli, Flavius D. Raslau, Oscar L. Lopez, MaryAnn Oakley, Donna M. Simpson, Anton P. Porsteinsson, Kim Martin, Nancy Kowalski, Melanie Keltz, Bonnie S. Goldstein, Kelly M. Makino, M. Saleem Ismail, Connie Brand, Gaby Thai, Aimee Pierce, Beatriz Yanez, Elizabeth Sosa, Megan Witbracht, Brendan Kelley, Trung Nguyen, Kyle Womack, Dana Mathews, Mary Quiceno, Allan I. Levey, James J. Lah, Ihab Hajjar, Janet S. Cellar, Jeffrey M. Burns, Russell H. Swerdlow, William M. Brooks, Daniel H. S. Silverman, Sarah Kremen, Liana Apostolova, Kathleen Tingus, Po H. Lu, George Bartzokis, Ellen Woo, Edmond Teng, Neill R. Graff-Radford, Francine Parfitt, Kim Poki-Walker, Martin R. Farlow, Ann Marie Hake, Brandy R. Matthews, Jared R. Brosch, Scott Herring, Christopher H. van Dyck, Adam P. Mecca, Susan P. Good, Martha G. MacAvoy, Richard E. Carson, Pradeep Varma, Howard Chertkow, Susan Vaitekunis, Chris Hosein, Sandra Black, Bojana Stefanovic, Chris Heyn, Ging-Yuek Robin Hsiung, Ellen Kim, Benita Mudge, Vesna Sossi, Howard Feldman, Michele Assaly, Elizabeth Finger, Stephen Pasternak, Irina Rachinsky, Andrew Kertesz, Dick Drost, John Rogers, Ian Grant, Brittanie Muse, Emily Rogalski, Jordan Robson, M.-Marsel Mesulam, Diana Kerwin, Chuang-Kuo Wu, Nancy Johnson, Kristine Lipowski, Sandra Weintraub, Borna Bonakdarpour, Nunzio Pomara, Raymundo Hernando, Antero Sarrael, Howard J. Rosen, Bruce L. Miller, David Perry, Raymond Scott Turner, Kathleen Johnson, Brigid Reynolds, Kelly MCCann, Jessica Poe, Reisa A. Sperling, Keith A. Johnson, Gad A. Marshall, Christine M. Belden, Alireza Atri, Bryan M. Spann, Kelly A. Clark, Edward Zamrini, Marwan Sabbagh, Ronald Killiany, Robert Stern, Jesse Mez, Neil Kowall, Andrew E. Budson, Thomas O. Obisesan, Oyonumo E. Ntekim, Saba Wolday, Javed I. Khan, Evaristus Nwulia, Sheeba Nadarajah, Alan Lerner, Paula Ogrocki, Curtis Tatsuoka, Parianne Fatica, Evan Fletcher, Pauline Maillard, John Olichney, Charles DeCarli, Owen Carmichael, Vernice Bates, Horacio Capote, Michelle Rainka, Michael Borrie, T-Y Lee, Rob Bartha, Sterling Johnson, Sanjay Asthana, Cynthia M. Carlsson, Allison Perrin, Anna Burke, Douglas W. Scharre, Maria Kataki, Rawan Tarawneh, Brendan Kelley, David Hart, Earl A. Zimmerman, Dzintra Celmins, Delwyn D. Miller, Laura L. Boles Ponto, Karen Ekstam Smith, Hristina Koleva, Hyungsub Shim, Ki Won Nam, Susan K. Schultz, Jeff D. Williamson, Suzanne Craft, Jo Cleveland, Mia Yang, Kaycee M. Sink, Brian R. Ott, Jonathan Drake, Geoffrey Tremont, Lori A. Daiello, Jonathan D. Drake, Marwan Sabbagh, Aaron Ritter, Charles Bernick, Donna Munic, Akiva Mintz, Abigail O’Connelll, Jacobo Mintzer, Arthur Wiliams, Joseph Masdeu, Jiong Shi, Angelica Garcia, Marwan Sabbagh, Paul Newhouse, Steven Potkin, Stephen Salloway, Paul Malloy, Stephen Correia, Smita Kittur, Godfrey D. Pearlson, Karen Blank, Karen Anderson, Laura A. Flashman, Marc Seltzer, Mary L. Hynes, Robert B. Santulli, Norman Relkin, Gloria Chiang, Michael Lin, Lisa Ravdin, Athena Lee, Ron Petersen, Thomas Neylan, Jordan Grafman, Tom Montine, Ronald Petersen, Lindsey Hergesheimer, Sarah Danowski, Catherine Nguyen-Barrera, Jacqueline Hayes, Shannon Finley, Michael Donohue, Matthew Bernstein, Matt Senjem, Chad Ward, Stephanie Rossi Chen, Robert A. Koeppe, Norm Foster, Tatiana M. Foroud, Steven Potkin, Li Shen, Kelley Faber, Sungeun Kim, Kwangsik Nho, Kristi Wilmes, Bryan M. Spann, Helen Vanderswag, Adam Fleisher, Ajay Sood, Kimberly S. Blanchard, Debra Fleischman, Konstantinos Arfanakis, Daniel Varon, Maria T. Greig, Bonnie Goldstein, Kimberly S. Martin, Gaby Thai, Aimee Pierce, Christopher Reist, Beatriz Yanez, Elizabeth Sosa, Megan Witbracht, Carl Sadowsky, Walter Martinez, Teresa Villena, Howard Rosen, Gad Marshall, Sheeba Nadarajah, Elaine R. Peskind, Eric C. Petrie, Gail Li, Jerome Yesavage, Joy L. Taylor, Steven Chao, Jaila Coleman, Jessica D. White, Barton Lane, Allyson Rosen, Jared Tinklenberg, Gloria Chiang, Scott Mackin, Rema Raman, Gustavo Jimenez-Maggiora, Devon Gessert, Jennifer Salazar, Caileigh Zimmerman, Sarah Walter, Olusegun Adegoke, Payam Mahboubi, Erin Drake, Mike Donohue, Craig Nelson, David Bickford, Meryl Butters, Michelle Zmuda, Bret Borowski, Jeff Gunter, Matt Senjem, Kejal Kantarci, Chad Ward, Denise Reyes, Kelley M. Faber, Kelly N. Nudelman, Yiu Ho Au, Kelly Scherer, Daniel Catalinotto, Samuel Stark, Elise Ong, Dariella Fernandez, Michelle Zmuda

**Affiliations:** 1 Department of Neurology and Institute of Neurology, Huashan Hospital, State Key Laboratory of Medical Neurobiology and MOE Frontiers Center for Brain Science, Shanghai Medical College, Fudan University, Shanghai, China; 2 Department of PET Center, Huashan Hospital, Fudan University, Shanghai, China; 3 Department of Shanghai Institute for Advanced Communication and Data Science, Shanghai University, Shanghai, China; 4 Department of Neurology, Qingdao Municipal Hospital, Qingdao University, Qingdao, China; 5 University of California, San Francisco, San Francisco, CA USA; 6 University of Southern California, Los Angeles, CA USA; 7 Mayo Clinic, Rochester, Rochester, NY USA; 8 University of California, Berkeley, Berkeley, CA USA; 9 University of Pennsylvania, Philadelphia, PA USA; 10 University of California, Davis, CA USA; 11 BWH/HMS, Boston, MA USA; 12 Indiana University, Bloomington, IN USA; 13 Washington University St. Louis, St. Louis, MO USA; 14 Alzheimer’s Association, Chicago, IL USA; 15 National Institute of Mental Health, Rockville, MD USA; 16 Rush University, Chicago, IL USA; 17 NIA, Bethesda, MD USA; 18 University of California, San Diego, San Diego, CA USA; 19 Denali Therapeutics, South San Francisco, CA USA; 20 NIH, Bethesda, MD USA; 21 Massachusetts General Hospital, Boston, MA USA; 22 Biogen, Cambridge, MA USA; 23 University of Wisconsin, Madison, WI USA; 24 Eli Lilly, Indianapolis, IN USA; 25 NCIRE/The Vererans Health Research Institute, San Francisco, CA USA; 26 Mayo Clinic, Scottsdale, AZ USA; 27 University College London, London, UK; 28 University of Southern California School of Medicine, Los Angeles, CA USA; 29 University of Michigan, Ann Arbor, MI USA; 30 Banner Alzheimer’s Institute, Phoenix, AZ USA; 31 University of Pittsburgh, Pittsburgh, PA USA; 32 Perelman School of Medicine, University of Pennsylvania, Philadelphia, PA USA; 33 Indiana University School of Medicine, Indianapolis, IN USA; 34 UPenn School of Medicine, Philadelphia, PA USA; 35 NCRAD/Indiana University School of Medicine, Indianapolis, IN USA; 36 Prevent Alzheimer’s Disease, 2020 Rockville, MD USA; 37 National Institute on Aging, Bethesda, MD USA; 38 Oregon Health & Science University, Portland, OR USA; 39 University of California—San Diego, San Diego, CA USA; 40 Baylor College of Medicine, Houston, TX USA; 41 Columbia University Medical Center, New York, NY USA; 42 University of Alabama—Birmingham, Birmingham, AL USA; 43 Mount Sinai School of Medicine, New York, NY USA; 44 Rush University Medical Center, Chicago, IL USA; 45 Wien Center, Miami Beach, FL USA; 46 Johns Hopkins University, Baltimore, MD USA; 47 University of South Florida: USF Health Byrd Alzheimer’s Institute, Tampa, FL USA; 48 New York University, New York, NY USA; 49 Duke University Medical Center, Durham, NC USA; 50 University of Kentucky, Lexington, KY USA; 51 University of Rochester Medical Center, New York, NY USA; 52 University of California Irvine IMIND, Irvine, CA USA; 53 University of Texas Southwestern Medical School, Dallas, TX USA; 54 Emory University, Atlanta, GA USA; 55 University of Kansas, Medical Center, Kansas City, KS USA; 56 University of California, Los Angeles, Los Angeles, CA USA; 57 Mayo Clinic, Jacksonville, Jacksonville, FL USA; 58 Yale University School of Medicine, New Haven, CT USA; 59 McGill University, Montreal-Jewish General Hospital, Montréal, QC Canada; 60 Sunnybrook Health Sciences, Ontario, Toronto Canada; 61 U.B.C. Clinic for AD & Related Disorders, Vancouver, Canada; 62 St. Joseph’s Health Care, Petaluma, CA USA; 63 Northwestern University, Evanston, IL USA; 64 Nathan Kline Institute, Orangeburg, CA USA; 65 Georgetown University Medical Center, Washington, DC USA; 66 Brigham and Women’s Hospital, Boston, MA USA; 67 Banner Sun Health Research Institute, Sun City, AR USA; 68 Boston University, Boston, MA USA; 69 Howard University, Washington, DC USA; 70 Case Western Reserve University, Cleveland, OH USA; 71 University of California, Davis—Sacramento, Sacramento, CA USA; 72 Dent Neurologic Institute, Orchard Park, NY USA; 73 Parkwood Institute, London, Canada; 74 Ohio State University, Columbus, OH USA; 75 Albany Medical College, Albany, NY USA; 76 University of Iowa College of Medicine, Iowa City, IA USA; 77 Wake Forest University Health Sciences, Winston Salem, NC USA; 78 Rhode Island Hospital, Providence, RI USA; 79 Cleveland Clinic Lou Ruvo Center for Brain Health, Las Vegas, NV USA; 80 Roper St. Francis Healthcare, Charleston, SC USA; 81 Houston Methodist Neurological Institute, Houston, TX USA; 82 Barrow Neurological Institute, Phoenix, AR USA; 83 Vanderbilt University Medical Center, Nashville, TN USA; 84 Long Beach VA Neuropsychiatric Research Program, Long Beach, CA USA; 85 Butler Hospital Memory and Aging Program, Providence, RI USA; 86 Neurological Care of CNY, East Syracuse, NY USA; 87 Hartford Hospital, Olin Neuropsychiatry Research Center, Hartford, CT USA; 88 Dartmouth-Hitchcock Medical Center, Lebanon, PA USA; 89 Cornell University, Ithaca, NY USA; 90 Rehabilitation Institute of Chicago, Feinberg School of Medicine, Northwestern University, Chicago, IL USA; 91 University of Washington, Seattle, WA USA; 92 University of Utah, Salt Lake City, UT USA; 93 UC Irvine, Irvine, CA USA; 94 NCRAD, Indianapolis, IN USA; 95 University of California, Irvine, Irvine, CA USA; 96 Premiere Research Inst (Palm Beach Neurology), West Palm Beach, FL USA; 97 Stanford University, Stanford, CA USA; 98 BWM/HMS, Boston, MA USA

**Keywords:** Neuroscience, Biomarkers

## Abstract

A biological research framework to define Alzheimer’ disease with dichotomized biomarker measurement was proposed by National Institute on Aging–Alzheimer’s Association (NIA–AA). However, it cannot characterize the hierarchy spreading pattern of tau pathology. To reflect in vivo tau progression using biomarker, we constructed a refined topographic ^18^F-AV-1451 tau PET staging scheme with longitudinal clinical validation. Seven hundred and thirty-four participants with baseline ^18^F-AV-1451 tau PET (baseline age 73.9 ± 7.7 years, 375 female) were stratified into five stages by a topographic PET staging scheme. Cognitive trajectories and clinical progression were compared across stages with or without further dichotomy of amyloid status, using linear mixed-effect models and Cox proportional hazard models. Significant cognitive decline was first observed in stage 1 when tau levels only increased in transentorhinal regions. Rates of cognitive decline and clinical progression accelerated from stage 2 to stage 3 and stage 4. Higher stages were also associated with greater CSF phosphorylated tau and total tau concentrations from stage 1. Abnormal tau accumulation did not appear with normal β-amyloid in neocortical regions but prompt cognitive decline by interacting with β-amyloid in temporal regions. Highly accumulated tau in temporal regions independently led to cognitive deterioration. Topographic PET staging scheme have potentials in early diagnosis, predicting disease progression, and studying disease mechanism. Characteristic tau spreading pattern in Alzheimer’s disease could be illustrated with biomarker measurement under NIA–AA framework. Clinical–neuroimaging–neuropathological studies in other cohorts are needed to validate these findings.

## Introduction

The neuropathological hallmarks of Alzheimer’s disease are the extracellular β-amyloid deposition and neurofibrillary tangles (NFTs) of intracellular misfolded phosphorylated tau (p-tau) protein. Unlike the diffuse distribution of amyloid plaques in the neocortex [1], the characteristic presence of NFTs indicated a hierarchical spreading pattern of tau pathology. In a landmark publication, Braak stages of tau pathology were proposed by Braak H and Braak E to illustrate how Alzheimer’s disease-related tau began in the transentorhinal cortex, then extended to the inferior and lateral temporal cortices, to the posterior cingulate cortex, and widely spread in isocortical cerebral areas in end-stage disease [2]. Replications of these findings were achieved in subsequent researches [3, 4], and eventually incorporated them into Alzheimer’s disease neuropathological criteria [5].

The recent advent of positron-emission tomography (PET) tau tracers enables tau pathology to be visualized, mapped, quantified, and examined in relation to cognition. Among the various tau PET ligands, flortaucipir (FTP; ^18^F-AV-1451) is the by far most widely studied one, selectively binding to paired helical filament tau within NFTs with high affinity [6]. Previously studies have demonstrated that flortaucipir retention has consistent patterns with the known neuropathological topology of NFTs [7] and that FTP signal is significantly related to cognition in both cognitively unimpaired and cognitively impaired individuals [8, 9].

The wide application of PET tracers and other cerebrospinal fluid (CSF) or plasma biomarkers in research has promoted the establishment of a classification framework of Alzheimer’s disease [10, 11]. In 2018, the National Institute on Aging–Alzheimer’s Association (NIA–AA) updated a research framework completely using objective biomarker measurement to define Alzheimer’s disease without clinical symptoms. This framework contributes to reflecting the biological nature of the disease, in which the status of β-amyloid plaque (labeled as A), of paired helical filament tau (labeled as T), and of neurodegeneration or neuronal injury (labeled as N) are dichotomized as normal or abnormal to determine the biomarker profiles [12]. However, the dichotomization of the tau biomarker failed to characterize the hierarchical spreading features of tau pathology. A more desirable approach would be establishing image staging schemes by examining both the quantity and locations of tau tracer retention in PET. This also offered practice in the definition of tau abnormality for identifying Alzheimer’s disease, for Alzheimer’s disease-related tau patterns could be summarized and separated from normal controls. To date, a few studies have been dedicated to developing such schemes to stage participants with tau PET, of which the common limitation is the lack of longitudinal clinical outcomes for various stages [13–15]. Unlike cross-sectional data focusing on measurement at a time, longitudinal data provide a more accurate method revealing differences by discovering different rates of clinical deterioration in long-term observation. Hence evaluating clinical trajectories in relation to tau stages using PET is critical for validating the clinical relevance of the staging scheme.

In this study, we first used one topographic staging scheme with flortaucipir PET to assign individuals into five stages. The primary goal was to describe and compare cognitive changes, clinical progressions, and biomarker profiles across stages. The longitudinally validated scheme may improve the precision of AD definition using A/T/N biomarkers and show the feasibility of predicting various disease progressions with in vivo tau imaging.

## Methods

The data used in this study were download from the online repository of Alzheimer’s Disease Neuroimaging Initiative (ADNI) (http://adni.loni.usc.edu/). The ADNI was launched in 2003 as a public–private partnership with the primary goal of testing whether serial magnetic resonance imaging, PET, and various clinical, biologic, and neuropsychological markers can be combined to measure the progression of mild cognitive impairment and early AD dementia. Each ADNI study site received approval from its institutional reviewed board. Written informed consent was obtained from all research participants. As flortaucipir scan was not performed in ADNI before 2015, the visit point of the initial flortaucipir scan was defined as the baseline.

### Participants

Individuals who underwent a flortaucipir scan with a contemporary clinical diagnosis of cognitive normal (CN), mild cognitive impairment (MCI), or dementia were included in our study. As a result, 734 participants were included in our study. Details of inclusion information are presented in Supplementary Fig. 1. Participants diagnosed with MCI were further classified into “Early MCI (EMCI)” and “Late MCI(LMCI)” based on the Wechsler Memory Scale-Revised (WMS-R) Logical Memory II story A score according to the ADNI criteria [16].

### PET imaging

Tau and amyloid PET imaging in the ADNI was performed using flortaucipir and florbetapir (^18^F-AV-45) separately. The imaging data downloaded from the ADNI dataset had been fully preprocessed using a standardized pipeline [17]. In brief, magnetic resonance imaging (MRI) T1-weighted magnetization prepared rapid acquisition gradient echo(MPRAGE) image obtained from each participant was first segmented and parcellated with Freesurfer (version 5.3.0) to establish a set of regions of interest (ROIs) in native space. Next, using SPM (version 5), the PET imaging was co-registered to the MPRAGE image which was collected at the same visit point, and the mean tracer uptake was calculated within each ROI. Intensity normalized standard uptake value ratio (SUVr) was generated by dividing regional tracer means by reference regions which were defined with inferior cerebellum gray matter for tau PET and whole cerebellum for amyloid PET. Composite SUVr of meta-ROI was calculated in a volume-weighted approach. Specifically, three composite SUVr of ROIs (Braak I/II ROI, Braak III/IV ROI, and Braak V/VI ROI) was generated which approximated the anatomical definitions of Braak stages I/II (transentorhinal stages), Braak stages III/IV (limbic stages), and Braak stages V/VI (neocortical stages) [18]. FreeSurfer regions that made up each Braak composite can be found in Supplementary Table 1. Values from four cortical gray matter regions (frontal, anterior cingulate, precuneus, and parietal cortex) were averaged to estimate the global florbetapir SUVr and a cutoff of 1.11 was used to determine amyloid abnormal (A + ) and normal (A−) [19]. Considering that each individual may have multiple florbetapir scans, only individuals with all scans showing under-cutoff global SUVrs are classified as amyloid normal. To reduce the contamination from regions where the off-target binding was frequently observed, flortaucipir data only with partial volume correction (PVC) were included in the analysis.

### Tau staging

We assigned participants into five stages based on the composite SUVrs of Braak ROIs at the first flortaucipir scan (Supplementary Fig. 2). In brief, participants with Braak V/VI ROI SUVr >1.873 were firstly assigned to the highest stage (stage 4). Second, the remaining participants with Braak III/VI ROI SUVr >1.523 were classified into stage 3. Next, participants with Braak III/VI ROI SUVr >1.307 fell into the intermediate (stage 2) and then Braak I/II ROI SUVr > 1.129 into stage 1. Lastly, those who remained were included in stage 0 as their Braak I/II ROI SUVr ≤ 1.129.

This staging strategy and its thresholds mainly referred to a four-level Braak ROI-based staging approach proposed by Schöll et al. and Maass et al. [13, 18]. In the original work, a conditional inference tree was employed to classify subjects with regard to their clinical diagnosis (i.e., young controls, older cognitively normal controls, Alzheimer’s disease). An SUVr threshold in Braak V/VI ROI was first derived with the whole sample entering the model. The participants above this threshold were classified as the highest stage. After the removal of those participants, the staging and threshold-deriving procedure continued with the next Braak ROI (III/IV). Continuing this approach, three thresholds could be obtained and those reaching no threshold were defined as the lowest stage. More details in the generation of the thresholds could be found in their work.

We initially applied the thresholds (Braak V/VI ROI SUVr >1.873, Braak III/VI ROI SUVr >1.307, Braak I/II ROI SUVr > 1.129) in our data to classify participants into four groups. However, the result showed a predominantly large sample size in the group of Braak V/VI ROI SUVr ≤1.873 and Braak III/VI ROI SUVr >1.304 (Supplementary Fig. 3). We suspected intergroup heterogeneity and thus further stratified the individuals with an additional threshold (Braak III/VI ROI SUVr >1.523) generated by the mixture modeling method [20]. This also creates more balanced sample sizes across stages for subsequent analyses.

### Measurement of CSF biomarkers and plasma NFL

CSF was collected from Lumbar punctures (LPs) in a standardized procedure as described in the ADNI procedures manual (http://adni.loni.usc.edu/). Samples were properly centrifuged, aliquoted to 500 μL in polypropylene tubes, frozen within 1 h after collection, shipped overnight on dry ice to the ADNI Biomarker Core laboratory, and stored at −80 °C. Aβ42, t-tau, and p-tau were measured with the corresponding Elecsys immunoassays on the Elecsys cobas e 601 analyzer as previously described [21]. A cutoff of 1098 pg/ml for CSF Aβ42 [22] was used to define amyloid positivity if the florbetapir scan was not available for the individual. Blood samples were also collected, processed, aliquoted, and frozen at −80 °C according to standardized procedures. Plasma neurofilament light chains (NFL) were analyzed using the ultrasensitive Single Molecule Array (Simoa) technique as previously described [23]. The measuring results within 2-year interval were included in the analysis at baseline.

### Measurement of cognition

Mini-mental state examination (MMSE) was used to evaluate global cognition while composite measures developed by ADNI were also used to reflect the memory (ADNI-MEM) and executive function (ADNI-EF) [24, 25]. The composite measure was generated with a part of RAVLT, ADAS-Cog, Logical Memory, and MMSE for ADNI-MEM and a part of Category Fluency, Trails A and B, Digit span backward, WAIS-R Digit Symbol Substitution, and 5 Clock Drawing items for ADNI-EF. Cognitive data acquired before and after the first flortaucipir scan were both included in longitudinal analyses.

### Statistical analyses

Baseline differences between stages were assessed using tests appropriate for the distribution of each variable and included ANOVA, Kruskal–Wallis, chi-square, or Fisher’s exact test. A test for linear trend across stages was conducted for variables that did not show significant results between stages.

Linear mixed-effects models were used to assess how cognition change over time across different tau stages. Rates of cognition change were estimated via the interaction between time and predefined group. In the first model, only the tau stage interacting with time was included in the model. In addition, in a second model, an interaction between tau stage, β-amyloid status, and time was included to investigate how the effect of the tau stage was potentially affected by the β-amyloid status. All the linear mixed-effects models in analyses included participant-specific random intercepts and slopes. We also included as covariates age at baseline, gender, years of education, and APOE ε4 counts in all models. MMSE was log-transformed so that estimated change could be interpreted on an annual percentage scale. Estimates and 95% CIs (confidence intervals) were based on 10,000-iteration parametric bootstrapping of the fitted models.

To assess the risk of clinical progression in the CN group and in the MCI group, unadjusted Kaplan–Meier plots were separately constructed. Clinical Dementia Rate–Global Score (CDR-GS) of 0.5 or greater was determined as endpoint event for the CN participants. The endpoint event for the MCI group was progressive cognitive deterioration [26], defined as the diagnosis of dementia, MMSE ≤ 24 at last visit or difference of MMSE ≥ 4 between the baseline and the last visit. In addition, we ran multivariate Cox proportional hazards models adjusted for age, gender, years of education, and APOE ε4 to compare the progression rates between stages.

All statistical analyses were performed using the R statistical software (version 3.5.1). Two-sided *P* values less than 0.05 were considered statistically significant.

## Results

We included 734 participants in our study (see the flow chart of study participants in Supplementary Fig. 1). The mean (SD, standard deviation) age of all the participants was 73.9 (7.69) years; 51.1% were women; 98.8% had more than 12 years of education; 37.2% had at least one APOE ε4 allele.

**Fig. 1:**
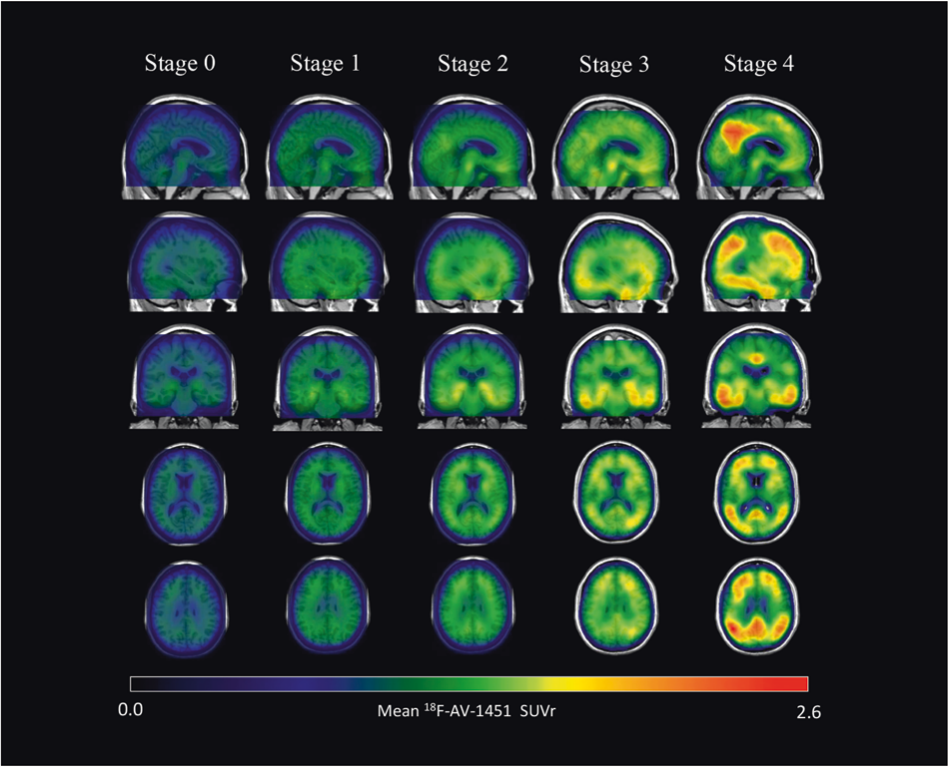
Parametric ^18^F-AV-1451 images across stages. In general, ^18^F-AV-1451 SUVr increased throughout the cortex and subcortex from stage 0 to stage 4 (numerical values shown in Table 1). Participants in stage 0 had tau levels corresponding to those of normal young adults. A dominating tau elevation in medial temporal regions (Braak I/II ROIs) was shown in stage 1. While stage 2 presented increased SUVrs in extra-medial temporal regions, stage 3 showed greater SUVrs increase in Braak III/IV ROIs including inferior and lateral temporal lobes. Stage 4 had significantly elevated ^18^F-AV-1451 SUVr extending into the neocortex. ROI region of interest, SUVr standard uptake value ratio.

The demographic, clinical, imaging, and CSF biomarkers characteristics of the included participants by tau stage are shown in Table 1, by amyloid status and tau stage in Supplementary Table 2. Of the 734 participants included, 48 (6.5%) participants were classified as stage 4, 134 (18.3%) as stage 3, 396 (54.0%) as stage 2, 81 (11.0%) as stage 1, and 75 (10.2%) as stage 0 (see staging flow chart in Supplementary Fig. 3). In general, tau burden increased significantly across various regions from stage 0 to stage 4 (Table 1 and Fig. 1).

**Table 1:** Baseline characteristics by Tau stage.

	Stage 0	Stage 1	Stage 2	Stage 3	Stage 4	Group test *P* value	Pairwise difference
No.	75 (10.2)	81 (11.0)	396 (54.0)	134 (18.3)	48 (6.54)	/	/
Age	71.0 (8.59)	72.1 (6.63)	74.0 (7.48)	77.2 (7.17)	71.8 (7.84)	<0.001	3 versus 1, 2, 4
Gender (female)	36 (48.6)	39 (47.6)	203 (51.3)	70 (52.2)	27 (56.3)	0.983	/
Years of education	16.5 (2.37)	16.7 (2.59)	16.7 (2.53)	16.2 (2.42)	15.6 (2.16)	0.017	4 versus 2
APOE ε4 non-carriers	40 (66.7)	43 (64.2)	240 (70.4)	46 (46.5)	9 (25.7)	<0.001	4 versus 0, 1, 2, 3; 3 versus 0, 1, 2
Clinical diagnosis							
CN	53 (71.6)	63 (76.8)	264 (66.7)	38 (28.4)	4 (8.30)	<0.001	
MCI	20 (27.0)	15 (18.3)	119 (30.1)	65 (48.5)	16 (33.3)		4 versus 0, 1, 2, 3; 3 versus 0, 1, 2
Dementia	1 (1.4)	4 (4.9)	13 (3.3)	31 (23.1)	28 (58.3)		
MMSE	28.8 (1.31)	28.4 (1.36)	28.7 (1.64)	26.5 (3.44)	22.7 (4.80)	<0.001	4 versus 0, 1, 2, 3; 3 versus 0, 1, 2
Memory composite	0.91 (0.64)	1.01 (0.68)	0.84 (0.63)	0.10 (0.77)	−0.64 (0.98)	<0.001	4 versus 0, 1, 2, 3; 3 versus 0, 1, 2
EF composite	1.06 (0.90)	1.17 (0.80)	0.88 (0.91)	0.33 (0.97)	−0.92 (1.25)	<0.001	4 versus 0, 1, 2, 3; 3 versus 0, 1, 2; 2 versus 1
Amyloid abnormal	27 (42.2)	27 (39.7)	165 (49.1)	95 (84.1)	43 (100.0)	<0.001	4 versus 0, 1, 2, 3; 3 versus 0, 1, 2; 2 versus 1
Aβ PET	1.08 (0.14)	1.06 (0.12)	1.11 (0.18)	1.34 (0.25)	1.49 (0.20)	<0.001	4 versus 0, 1, 2, 3; 3 versus 0, 1, 2;
Tau PET							
Braak I/II ROI	1.05 (0.06)	1.24 (0.14)	1.31 (0.18)	1.74 (0.35)	2.07 (0.52)	<0.001	All paris
Braak III/IV ROI	1.22 (0.06)	1.26 (0.04)	1.40 (0.06)	1.73 (0.20)	2.55 (0.70)	<0.001	All paris
Braak V/VI ROI	1.31 (0.09)	1.32 (0.07)	1.45 (0.08)	1.64 (0.12)	2.50 (0.67)	<0.001	4 versus 0, 1, 2, 3; 3 versus 0, 1, 2; 2 versus 1,0
CSF Aβ42 (pg/mL)	1327.6 (540.3)	1428.9 (715.9)	1332 (642.6)	888.9 (624.4)	606.4 (381.3)	<0.001	4 versus 0, 1, 2; 3 versus 0, 1, 2
CSF p-tau (pg/mL)	19.9 (7.18)	18.1 (5.76)	21.4 (9.27)	34.9 (15.8)	37.2 (17.8)	<0.001	4 versus 0, 1, 2; 3 versus 0, 1, 2
CSF t-tau (pg/mL)	227.0 (69.1)	203.7 (62.4)	237.7 (86.8)	345.9 (130.2)	381.3 (184.8)	<0.001	4 versus 0, 1, 2; 3 versus 0, 1, 2
Plasma NFL (pg/mL)	34.2 (14.3)	35.7 (24.5)	38.1 (20.0)	41.6 (16.1)	51.6 (19.5)	0.095	/

*Aβ* β amyloid, *CN* cognitively normal, *EF* executive function, *MCI* mild cognitive impairment, *MMSE* mini-mental status examination, *NFL* neurofilament light chain, *p-tau* phosphorylated tau, *t-tau* total tau.

Continuous variables are expressed as mean (SD) and categorical variables as number (%). Braak I through VI labels represent composite regions corresponding to Braak neurofibrillary tangle stages. For group tests with *P* < 0.05, unadjusted pairwise post hoc differences are reported.

### Distribution of stages in four clinical diagnostic groups

**Fig. 2:**
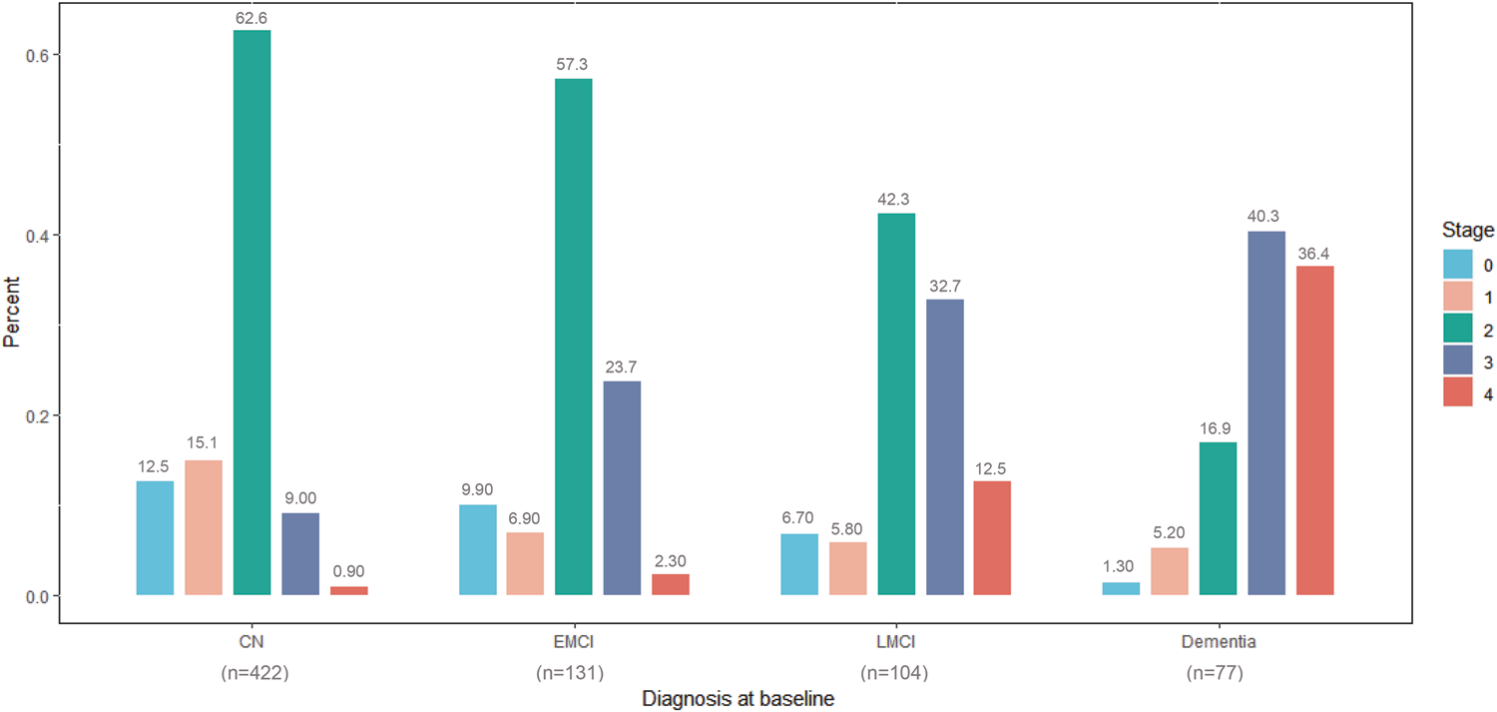
Distribution of different tau stages across clinical diagnostic groups. Under the horizontal axis are numbers of included participants in four diagnostic groups. Proportions of low stages and intermediate stages (stage 0, 1, 2) decreased with clinical deterioration, while proportions of high stages (stage 3, 4) increased. CN cognitively normal, EMCI early mild cognitive impairment, LMCI late mild cognitive impairment.

The proportion of stage 2 (62.6%) was highest in the CN group compared with those of other stages. Stage 2 (57.3%) or stage 3 (23.7%) accounted for the most part of the EMCI group. Most participants with LMCI were seen in stage 2 (42.3%) or stage 3 (32.7%), while most diagnosed with dementia were classified in stage 3 (40.3%) or stage 4 (36.4%). There was a decreasing pattern among stage 0, stage 1, and stage 2 and an increasing pattern among stage 3 and stage 4 when the clinical diagnosis became more severe (Fig. 2).

### Demographic, clinical, and PET imaging characteristics at baseline

All stages had comparable gender ratios but differed by age, years of education, and APOE ε4 counts. Dementia, MCI, and CN were predominant in stage 4, stage 3, and stage 0, 1, or 2, respectively. The diagnostic profile across stages among participants with abnormal amyloid was similar to the whole sample. Most MCI participants with abnormal amyloid were seen in stage 3 (Supplementary Table 2).

Overall, stage 0, 1, and 2 had similar cognitive levels at baseline (*P* > 0.05 for all pairwise comparisons for these three stages in terms of MMSE and memory composite), which were significantly better compared with stage 3 and stage 4. Participants in stage 4 had the worst performance on all three cognition measurements. Meanwhile, stage 4 also had the highest florbetapir SUVr, followed by stage 3 and stage 2, 1, or 0. Intriguingly, all the participants in stage 4 were amyloid positive. In total, 84.1% of the participant in stage 3 with positive amyloid status were observed, which was significantly different from the proportions observed in stages 0, 1, or 2.

### Biomarkers in CSF and plasma

**Fig. 3:**
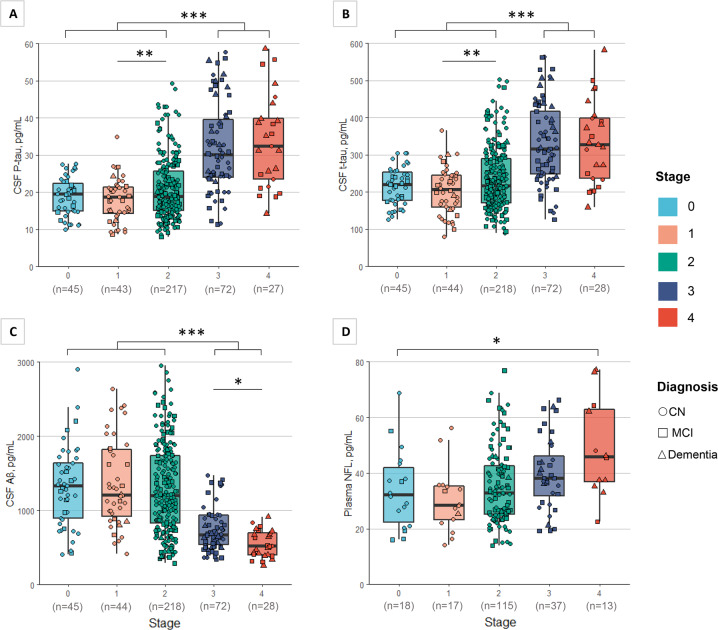
Baseline CSF biomarkers and plasma NFL profiles across tau stages. **A** CSF p-tau across stages. **B** CSF t-tau across stages: CSF p-tau/t-tau levels were significantly higher for stage 3 and stage 4 respectively compared with stages 0, 1, or 2. Stage 2 significantly differed from stage 1. **C** CSF Aβ levels across stages: CSF Aβ levels were significantly lower for stage 3 and stage 4 respectively compared with stage 0, 1, or 2. Stage 4 marginally differed from stage 3 (*P* = 0.057). **D** Plasma NFL levels across stages: No significant difference was detected among stages for plasma NFL(*P* = 0.095). Under the horizontal axes are numbers of included participants in comparison. CN cognitively normal, MCI mild cognitive impairment, NFL neurofilament light chain, p-tau phosphorylated tau, t-tau total tau. *P < 0.1; **P < 0.05; ***P < 0.005.

Individuals in stage 3 and stage 4 respectively had significantly higher CSF p-tau levels than individuals in stage 0, 1, or 2, while no significant difference was found between stage 3 and stage 4 (Fig. 3A). CSF p-tau level in stage 2 was also significantly higher compared with stage 1. These results were the same across stages for CSF t-tau (Fig. 3B). In terms of CSF Aβ42 levels, stage 3 and stage 4 were significantly lower when compared with stage 0, 1, or 2, and stage 4 marginally differed from stage 3 (*P* = 0.057) (Fig. 3C). As we do not detect significant change among stages for plasma NFL (Table 1, *P* = 0.095), a dose-response trend was examined and a significant result was identified with higher plasma NFL levels for higher stages (*P* for trend = 0.008) (Fig. 3D).

### Longitudinal cognition in each tau stage

**Fig. 4:**
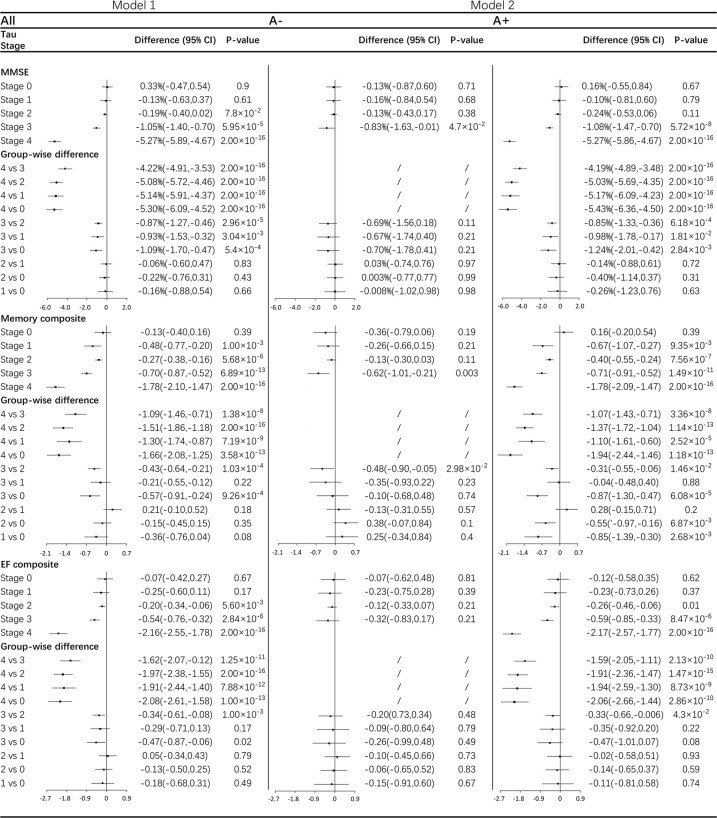
Cognitive changes and comparisons across stages based on linear mixed-effects models. Analyses of cognitive change were adjusted for age, gender, education years and ApoE ε4 counts. In both models, rates of cognitive changes with group-wise comparisons are expressed as % per year for MMSE and 10^−1^ per year for Memory or EF composite with 95%CI. The numbers of participants included and comparisons between the A+ and A− within the same stage are shown in Supplementary Table 3 and Supplementary Table 4 for each analysis. A+ abnormal β-amyloid, A− normal β-amyloid, CI confidence interval, EF executive function, MMSE mini-mental state examination.

**Fig. 5:**
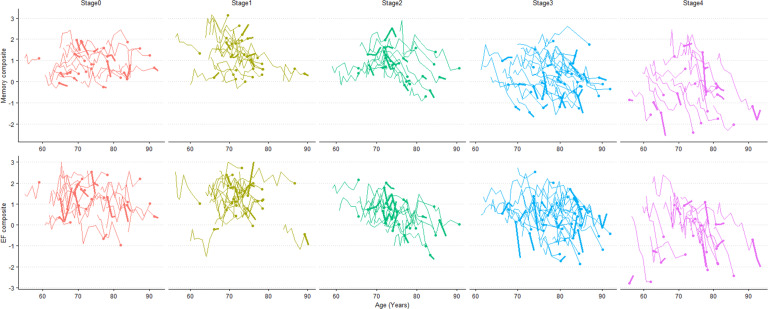
Trajectories of memory and executive function versus age by tau stage. Each line represents one participant’s trajectory, with the dot indicating the baseline, the thinner part of the line indicating the measures before the baseline, and the thicker part of the line indicating measures after the baseline. Participants showed a perceivable decline in stage 1, 2, 3, or 4 for memory composite and in stage 2, 3, or 4 for EF composite. The differences in rates of cognitive decline between stages were characterized using linear mixed-effects models (results shown in Fig. 4). For the panel of stage 2, a random subset of 20% of the data is shown to reduce overlap in the lines. EF executive function.

Potential cognitive changes and cognitive trajectory differences between stages were investigated by linear mixed-effects models (Fig. 4) and by plotting composites versus age stratified by stage (Fig. 5). The numbers of participants included in linear mixed-effects models for different cognitive measures are shown in Supplementary Table 3. Across five stages in all participants, significant declines were observed in stages 1, 2, 3, and 4 for memory composite (*P* ≤ 0.001 for all four stages), in stages 2, 3, and 4 for EF composite (*P* = 0.006 for stage 2 and *P* < 0.001 for stage 3 and stage 4), and in stage 3 and stage 4 for MMSE score (both *P* < 0.001). Compared with other stages, stage 4 always showed accelerated deterioration for all three cognitive measures (all *P* < 10^−7^). Participants assigned in stage 3 also showed faster rates of cognitive decline than those in stage 2 (*P* values ranging from 2.96 × 10^−5^ to 1.00 × 10^−3^) and in stage 0 (*P* values ranging from 1.00 × 10^−3^ to 0.02). While rates of MMSE change differed between stage 3 and stage 1 (*P* = 3.04 × 10^−3^), both stages have comparable rates of memory (*P* = 0.22) and EF (*P* = 0.17) decline. No significant differences were found in rates of cognitive change between stages 0, 1, and 2 (all *P* values of group-wise difference >0.05).

These results similarly applied to the participants with abnormal amyloid status only except for the memory composite where stage 2 and stage 1 became significantly different from stage 0. Significant differences were remarkably less seen among individuals with normal amyloid status. Only stage 3 showed significant changes in MMSE score and memory composite. The significant group-wise difference was only seen between stage 2 and stage 3 for memory composite. Participants with abnormal amyloid in stage 3 showed a significantly slower memory decline than those with normal amyloid (Supplementary Table 4).

### Clinical progression for each tau stage

**Fig. 6:**
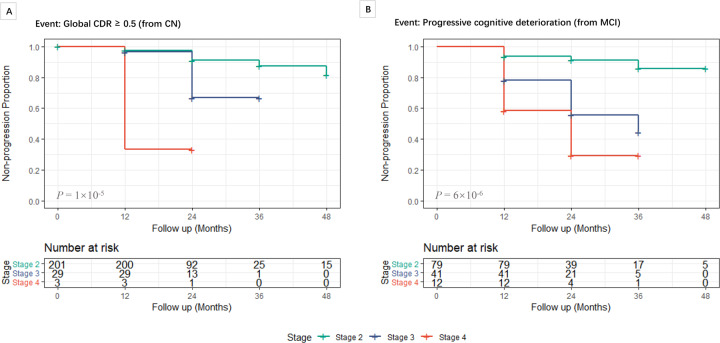
Kaplan–Meier curves showing the cumulative probability of clinical progression. Clinical progression was shown for each tau stage in CN (**A**) and MCI (**B)** group. Progressive cognitive deterioration defined as (1) diagnosis of dementia or (2) MMSE ≤24 at last visit or (3) difference of MMSE ≥4 between the first visit and the last visit. Results of the log-rank test showed a significant difference between stages. Stage 0 and stage 1 were not included in analyses for no events occurred in follow-up. CN cognitively normal, MCI mild cognitive impairment, CDR clinical dementia rating.

A Kaplan–Meier analysis and the corresponding log-rank test are exhibited in Fig. 6. As no event occurred in stage 0 or stage 1 for both diagnostic groups, they were not included in analyses. The results of multivariate Cox regression analyses were shown in Table 2. We found that individuals in stage 3 (HR (95% CI) = 3.29 (1.09, 9.97), *P* = 3.53 × 10^−2^) and stage 4 (HR (95% CI) = 18.7 (3.46, 100.9), *P* = 6.64 × 10^−4^) had an increased risk of conversion to CDR-GS of 0.5 or greater compared with individuals in stage 2. CN individuals in stage 4 (HR (95% CI) = 4.99 (0.96, 25.8), *P* = 5.53 × 10^−2^) had a marginally increased risk of conversion to GDR-GS ≥ 0.5 compared with stage 3.

**Table 2:** Progression risk from CN and MCI.

Stage	Hazard ratio (95% CI)	*P* value	Hazard ratio (95% CI)	*P* value
Progression to CDR-GS ≥ 0.5 from CN				
Stage 2	Reference	/	/	/
Stage 3	3.29 (1.09, 9.97)	3.53 × 10^−2^	Reference	/
Stage 4	18.7 (3.46,100.9)	6.64 × 10^−4^	4.99 (0.96, 25.8)	5.53 × 10^−2^
Progression to progressive cognitive deterioration from MCI				
Stage 2	Reference	/	/	/
Stage 3	4.23 (1.68,10.6)	2.20 × 10^−3^	Reference	/
Stage 4	8.99 (2.77,29.1)	2.54 × 10^−4^	1.92 (0.79, 4.68)	0.15

*CI* confidence interval, *CN* cognitively normal, *MCI* mild cognitive impairment, *CDR-GS* clinical dementia rating– global score.

Progressive cognitive deterioration defined as (1) diagnosis of dementia or (2) MMSE ≤ 24 at last visit or (3) difference of MMSE ≥ 4 between the first visit and the last visit.

Hazard ratios (95% CI) were calculated using Cox regression analyses and corrected for baseline age, gender, years of education and ApoE ε4 counts. Stage 0 and stage 1 were not included in analyses for no events occurred in follow-up.

In MCI patients, compared with stage 2, participants in stage 3 (4.23 (1.68, 10.6), *P* = 2.20 × 10^−3^) or stage 4 (HR (95% CI) = 8.99 (2.77, 29.1), *P* = 2.54 × 10^−4^) also had an elevated risk of progressive cognitive deterioration under Cox proportional hazards models. However, we did not detect differences in conversion risk among MCI individuals between stage 3 and stage 4 (HR (95% CI) = 1.92 (0.79, 4.68), *P* = 0.15).

## Discussion

In this study, we demonstrated a topographic PET staging scheme with longitudinal validation. Cognitive decline and clinical progression were distinct across stages and generally showing monotonically decreasing patterns from stage 0 to stage 4. The findings are congruent with the proposed temporal evolution of tau in Alzheimer’s disease and showed potential in early diagnosis of Alzheimer’s disease and discriminating different disease progressions.

The earliest cognitive decline was detected by memory composite in stage 1. More importantly, when participants with abnormal amyloid status were isolated from the full population, this became significantly different from stage 0 in which no significant longitudinal change of cognition was observed. Previous longitudinal and cross-sectional studies using continuous measures of tau pathology have shown that there was a relationship between flortaucipir binding in the media temporal lobe and episodic memory performance, even in CN individuals [27]. Since memory decline is regarded as a harbinger of future global cognitive deterioration in Alzheimer’s disease [28], this means that early increased tau deposit in regions of Braak I/II stages with abnormal amyloid status already can identify the individuals whose cognition starts to deteriorate. Thus, the SUVr threshold in Braak I/II ROI classifying stage 0 and stage 1 might be considered as the cutoff of tau biomarker to define Alzheimer’s disease. This point was further supported by more positive results from stage 2 when tau levels were elevated in extra-medial temporal regions. Both memory and EF composite exhibited significant change at this stage. Although stage 2 did not have a significantly faster rate of memory decline than stage 1, it was confirmed that the memory cognitive trajectory significantly distinguished from that of stage 0. The discrepancy was also ascertained in time-to-event analyses where no endpoint event occurred in stage 1 and stage 0. Compared with memory composite, the cognitive measure change and the group-wise difference became significant later in higher stages for MMSE and EF composite likely because the affection of other cognitive domains required tau pathology involvement in wider brain regions [8, 29]. Evidence from stage 1 and stage 2 was reinforced by the cross-sectional findings from high stages at baseline, where MMSE, memory, and EF composite all had significantly degraded performance, in addition to the high proportions of MCI and dementia. Worse clinical and cognitive profiles in stage 3 and stage 4 suggested that cognitive and clinical deterioration might have already begun in stage 2 or earlier. Incidentally, the mean CSF p-tau levels of stage 1 or stage 2 were approximate to or even lower than the known cutoff defining tau abnormality [30, 31]. Taken together, we supposed that the SUVr threshold in Braak I/II ROI classifying stage 0 and stage 1 could serve as a sensitive cutoff of tau biomarker in the definition of Alzheimer’s disease.

The topographic PET staging scheme is also valuable in predicting distinct clinical progression of the disease. A postmortem neuropathological study conducted by Qian et al. discovered that rates of the clinical and cognitive scores change varied depending on the Braak stage such that high Braak stage versus low Braak stage had additional cognition decrease per year [32]. Our analysis paralleled their results, showing that all the cognitive measures differed significantly between stage 2 and stage 3 and between stage 3 and stage 4. Furthermore, our Cox proportional hazard models also exhibited significantly faster progression rates of stage 4 and stage 3 than that of stage 2. The comparable progression rates between stage 3 and stage 4 likely resulted from a relatively short prospective visiting period. Unlike the longitudinal cognitive analyses, time-to-event analyses have a higher demand for the long prospective visiting period to detect a group-wise difference in advanced stages, for it did not include the individuals with dementia. From a clinical-neuroimaging view, our results add to the evidence validating in vivo PET staging with flortaucipir as a surrogate for the postmortem Braak stage.

Together with our findings on early diagnosis, the topographic PET staging scheme for tau pathology presents huge implications for clinical trials of Alzheimer’s disease. Participants with abnormal amyloid and stage 1 or higher could be listed as one of the inclusion criteria to start anti-tau agent, particularly for those trials aiming at early intervention. Moreover, participants could be stratified into more homogeneous groups, which is critical to improve the power of the trial and reduce the required sample size [32]. Besides, considering the close association between tau and neurodegeneration in Alzheimer’s disease [12], heterogeneity in cognitive trajectories and clinical progressions across tau stages also indicates that a single dichotomous classification of the neurodegeneration dimension is an oversimplification. Two levels (N + or N−) certainly cannot reflect diverse disease severity as the NIA–AA framework recommended [12]. Future studies with multilevel staging schemes may also be demanded characterizing neurodegeneration profiles under the A/T/N framework.

Staging tau pathology with topographic PET image also provides insights into tau pathology per se and its relationship with amyloid pathology. Albeit cross-sectional, nearly a quarter of CN individuals in stage 0 or stage 1 with positive amyloid status (Supplementary Table 2) in our study gave support that abnormal amyloid precedes even early stages (i.e., Braak I/II) of PET detectable tangle formation [33]. It is also worth noting that only amyloid abnormal participants were included in stage 4 which indicated that, for Alzheimer’s disease, wide presence of tau in the neocortex might be trigged by amyloid pathology [34]. This was the same with the findings in Schöll’s work which applied the same staging scheme with different thresholds to another cohort sample of smaller size [13]. Among participants without amyloid abnormality, the intermediate stage showing elevated tau levels in Braak III/IV regions was mostly seen in cognitively unimpaired participants. This profile supported the recent findings on normal aging with tau involvement in Braak I–IV regions [35], which may be designated as primary aging-related tauopathy (PART) [36].

Previous research suggested that mere presence of tau was not sufficient to cause cognitive changes [37]. However, in our analysis, a significant memory change was captured in normal amyloid participants of stage 3 after adjustment for age, which was significantly different from the stable memory condition in stage 2. Thus, we gave evidence that highly elevated tau pathology in Braak III/IV regions could independently result in cognitive decline in absence of amyloid pathology. In our analysis with model 2, we detected an amyloid-tau interaction signal on memory decline and then found the differed rates between normal amyloid and abnormal amyloid in stage 2. This implies that memory decline could be accelerated by β-amyloid interacting with tau and presents evidence to the point that Braak III/IV stages were a transition phase of evolving Alzheimer’s disease [18]. Through cognitive evaluation, Alzheimer’s disease might be distinguished from PART in stage 2 or even higher, for their cognitive trajectories separated at this point. Failure of discovering the interaction effect on cognition in previous tau studies [13, 38] and in our study when MMSE or EF composite used conveys the message that large sample size in each subgroup, longitudinal design and composite measures sensitive to early cognition change are required to show the weak interacting effect. Thus, for further analysis, a larger sample size is needed to detect the difference between A+ and A− in stage 3. Intriguingly, a newly publicized longitudinal study by Betthauser et al. also found an interaction effect between the two pathologies using eight-year PACC (preclinical Alzheimer’s cognitive composite) data, yet with a relatively small sample size [39]. It may attribute to the classification strategy in their work by which A and T profiles were divided to assign participants into four groups (A−T−, A−T+, A+T−, A+T+). All the abnormal tau individuals were grouped as a whole with no further staging, which was a remarkable difference from our study. The outcome difference would likely be exaggerated when comparing A−T+ and A+T+, for there was no tau pathology in stage 4 for A−T+ but for A+T+. It is an implication for future work examining amyloid effect on tau that the two comparing groups should be placed in the same tau conditions to produce a precise conclusion.

While the relation between amyloid and tau is under discussion, the relation between CSF p-tau and PET tau is either not firmly established at present. In our study, levels of CSF p-tau increased with ascending PET tau stage generally, which was consistent with a recent publication in which good linear association was shown between CSF p-tau and predefined-meta-ROI flortaucipir-PET uptake [40]. However, the correlation was not perfect. We did not observe a significant difference between the high stages (stage 3 and stage 4). A potential interpretation is that while tau continues accumulation reflected by PET tau, CSF p-tau seems to reach a plateau later in the disease [12, 41]. Meanwhile, we did not either observe a significant difference between the lowest stages (stage 0 and stage 1), which seemed to conflict with the current view that abnormal CSF p-tau preceded the abnormal PET tracer uptake. There was a fact discovered by Mattson et al. that >50% of the full population and 100% of the preclinical Alzheimer’s disease population had elevated flortaucipir uptake in Braak I/II ROI while CSF p-tau was still normal [42]. A high proportion of CSF-/PET+ discordant participants in stage 1 might explain this result. Another reason, we think, was likely the pre-analytical bias. Contamination by target-off binding near the hippocampus could not be adequately corrected by PVC, which was stated by ADNI in February 2020 (http://adni.loni.usc.edu/). This might lead to high SUVr in Braak I/II ROI, causing more participants originally in stage 0 assigned to stage 1. The concordance between the CSF p-tau and PET tau, especially in the early stage of the disease, needs further demonstration using the next generation tau tracers with less off-target binding [6] and with CSF p-tau of other kinds different from the ^181^p-tau we used herein.

Apart from the large sample size, long-period data, different cognitive evaluation measures, this work showed the advantage of strong clinical relevance at the beginning. As expected, an increasing proportion of high tau stage was seen in individuals with more severe clinical diagnoses. This distribution of stage profiles in four diagnostic categories was consistent with clinical-pathological findings [2] and with other tau staging studies using different algorithms [14]. Compared to the original staging scheme developed by Schöll M and his team, we made an improvement based on their work by further stratifying participants with elevated tau levels in Braak III/IV regions into stage 2 and stage 3. The widely detected differences between these two stages confirmed heterogeneity in the original group of Braak V/VI ROI SUVr ≤1.873 and Braak III/VI ROI SUVr >1.304 and illustrated that extra-medial temporal regions played a critical role in the development of Alzheimer’s disease. Different levels of tau pathology in these regions determined different fates of clinical progression, which should be fully recognized in disease tracking.

Despite these advantages, this study has several limitations. First, as tau PET was introduced in 2015 and long-term follow-up was not available for participants who have undergone flortaucipir-PET imaging, this length of the period from the first flortaucipir scan was not long enough to investigate the prospective clinical progression, especially for those in low stage or with normal amyloid status. For the same reason, we included retrospective data in the analysis of cognitive trajectories (Supplementary Table 5). Second, contamination by target-off binding that could not be adequately corrected by PVC in ADNI might lead to inaccurate SUVr estimation in Braak ROIs and biased staging. Third, this study is specific for the particular tau tracer used and it is not yet known whether these results will be replicated with tau tracers of other types. Fourth, ADNI has a relatively pure Alzheimer’s disease population by mainly including amnestic patients with high-level homogeneity in race and education. Thus, the sample is not representative of the population in the real world, and reproducibility of findings with different phenotypes of Alzheimer’s disease and different participants from other cohorts would be beneficial for the refinement of this staging scheme. Fifth, as we require only one amyloid PET scan out of multiple to be amyloid positive, it might be biased toward amyloid positive. Clinical–neuroimaging–neuropathological studies are also needed to further validate these findings.

In conclusion, we describe a topographic tau PET staging scheme that shows potentials in early diagnosis, predicting clinical progression, and studying disease mechanisms. Characteristic tau spreading pattern in Alzheimer’s disease could be demonstrated with biomarker measurement under the NIA–AA framework.

## Supplementary information

Supplementary materials

## Data Availability

Data used in this study were originally from the online repository of the Alzheimer’s Disease Neuroimaging Initiative (ADNI) database (http://adni.loni.usc.edu/), which is easily available for the research public. The data generated during processing and analyzing are available from the authors upon request.

## Code availability

All scripts used in analyses are available from the authors upon request.
