# Supplementary material for: Staging tau pathology with tau PET in Alzheimer’s disease: a longitudinal study
Source: Transl Psychiatry. 2021 Sep 18;11:483. doi: 10.1038/s41398-021-01602-5 (PMC8449785; doi:10.1038/s41398-021-01602-5)
Supplement: Supplementary file 1 — Supplementary materials [file 41398_2021_1602_MOESM1_ESM.pdf]

Supplementary materials

Content

Supplementary Table 1. Freesurfer-defined region codes for Braak ROIs 2
Supplementary Table 2. Baseline characteristics by amyloid status and tau Stage. 4
Supplementary Table 3. Numbers of participants included in linear mixed effects models 6
Supplementary Table 4. Comparisons of cognitive change between abnormal amyloid versus normal amyloid status 7
Supplementary Table 5. Distributions of time points in longitudinal analyses of cognition 8
Supplementary Figure 1. Flow chart of study participants........ 9
Supplementary Figure 2. Braak ROIs used for tau staging. 10
Supplementary Figure 3. Flow chart of in vivo staging using flotaucipir PET. 11

Supplementary Table 1. Freesurfer-defined region codes for Braak ROIs

Braak ROI	Code	Regions
**Braak 1 and 2 composite region (Braak12)**		
**Braak 1**	1006	L_entorhinal
	2006	R_entorhinal
**Braak 2**	17	L_hippocampus
	53	R_hippocampus
**Braak 3 and 4 composite region (Braak34):**		
**Braak 3**	1016	L_parahippocampal
	1007	L_fusiform
	1013	L_lingual
	18	L_amygdala
	2016	R_parahippocampal
	2007	R_fusiform
	2013	R_lingual
	54	R_amygdala
**Braak 4**	1015	L_middletemporal
	1002	L_caudantcing
	1026	L_rostantcing
	1023	L_postcing
	1010	L_isthmuscing
	1035	L_insula
	1009	L_inferiortemporal
	1033	L_temppole
	2015	R_middletemporal
	2002	R_caudantcing
	2026	R_rostantcing
	2023	R_postcing
	2010	R_isthmuscing
	2035	R_insula
	2009	R_inferiortemporal
	2033	R_temppole
	1028	L_superior_frontal
**Braak 5 and 6 composite region (Braak56)**		
**Braak 5**	1012	L_lateral_orbitofrontal
	1014	L_medial_orbitofrontal
	1032	L_frontal_pole
	1003	L_caudal_middle_frontal
	1027	L_rostral_middle_frontal

	1018	L_pars_opercularis
	1019	L_pars_orbitalis
	1020	L_pars_triangularis
	1011	L_lateraloccipital
	1031	L_parietalsupramarginal
	1008	L_parietalinferior
	1030	L_superiortemporal
	1029	L_parietalsuperior
	1025	L_precuneus
	1001	L_bankSuperiorTemporalSulcus
	1034	L_tranvtemp
	2028	R_superior_frontal
	2012	R_lateral_orbitofrontal
	2014	R_medial_orbitofrontal
	2032	R_frontal_pole
	2003	R_caudal_middle_frontal
	2027	R_rostral_middle_frontal
	2018	R_pars_opercularis
	2019	R_pars_orbitalis
	2020	R_pars_triangularis
	2011	R_lateraloccipital
	2031	R_parietalsupramarginal
	2008	R_parietalinferior
	2030	R_superiortemporal
	2029	R_parietalsuperior
	2025	R_precuneus
	2001	R_bankSuperiorTemporalSulcus
	2034	R_tranvtemp
**Braak 5**	1021	L_pericalcarine
	1022	L_postcentral
	1005	L_cuneus
	1024	L_precentral
	1017	L_paracentral
	2021	R_pericalcarine
	2022	R_postcentral
	2005	R_cuneus
	2024	R_precentral
	2017	R_paracentral

Supplementary Table 2. Baseline characteristics by amyloid status and tau Stage.

	A-				A+					
	**Stage0**	**Stage1**	**Stage2**	**Stage3**	**Stage0**	**Stage1**	**Stage2**	**Stage3**	**Stage4**	P value
**No.**	37	41	171	18	27	27	165	94	43	
**Age**	71.4(8.44)	72.4(5.61)	73.8(7.77)	75.8(7.35)	72.2(9.27)	72.5(7.21)	75.1(7.46)	78.3(7.00)	72.3(7.81)	< 0.001[J]
**Gender(Female)**	21(56.8)	19(46.3)	95(55.6)	9(50.0)	11(40.7)	15(55.6)	81(49.1)	50(52.6)	26(60.5)	0.93
**Years of education**	16.5(2.34)	16.5(2.89)	16.6(2.63)	17.2(1.89)	16.4(2.29)	16.9(2.38)	16.8(2.59)	15.9(2.52)	15.7(2.13)	0.08
**APOE ε4 non-carriers**	24(77.4)	30(83.3)	128(81.0)	13(92.9)	13(54.2)	11(44.0)	88(59.1)	32(40.0)	9(26.5)	< 0.001[I,γ,δ]
**Clinical diagnosis**										< 0.001[C,D,E,F,G,H,I,J]
CN	28(75.7)	33(80.5)	119(69.6)	8(44.4)	18(66.7)	23(85.2)	111(67.3)	29(30.5)	4(9.30)	
MCI	9(24.3)	7(17.0)	50(29.2)	9(50.0)	8(29.6)	2(7.40)	46(27.9)	44(46.3)	15(34.9)	
Dementia	0(0.00)	1(2.40)	2(1.20)	1(5.60)	1(3.70)	2(7.40)	2(7.4)	22(23.2)	24(55.8)	
**MMSE**	29.0(0.93)	28.9(1.22)	28.8(1.56)	27.7(2.54)	28.8(1.42)	28.8(1.58)	28.6(1.78)	26.4(3.42)	22.6(4.95)	< 0.001[C,D,E,F,G,H,I,J]
**Memory composite**	0.99(0.60)	0.94(0.64)	0.60(0.04)	0.55(0.78)	0.85(0.70)	1.12(0.80)	0.76(0.67)	0.08(0.72)	-0.61(0.97)	< 0.001[C,D,F,G,H,I,J]
**EF composite**	1.25(0.64)	1.08(0.78)	0.96(0.94)	0.97(0.82)	0.80(1.03)	1.29(0.73)	0.72(0.89)	0.25(0.96)	-0.90(1.25)	< 0.001[D,E,F,G,H,I,J]
**Aβ PET**	1.00(0.07)	1.00(0.06)	1.00(0.06)	0.98(0.04)	1.17(0.16)	1.12(0.13)	1.21(0.19)	1.40(0.22)	1.49(0.20)	< 0.001[C,D,E,F,G,H,I,α,β,γ,δ]
**Tau PET**										
Braak I/II ROI	1.05(0.06)	1.23(0.14)	1.28(0.16)	1.71(0.52)	1.04(0.06)	1.26(0.16)	1.35(0.19)	1.74(0.30)	2.09(0.51)	< 0.001[a,b,c,e,f,A,B,C,D,F,G,H,I,J,γ]
Braak III/IV ROI	1.23(0.06)	1.26(0.04)	1.40(0.06)	1.62(0.13)	1.22(0.06)	1.27(0.03)	1.40(0.06)	1.76(0.21)	2.59(0.73)	< 0.001[b,c,e,f,A,B,C,D,E,F,G,H,I,J,γ]
Braak V/VI ROI	1.32(0.09)	1.31(0.07)	1.46(0.07)	1.61(0.08)	1.30(0.08)	1.32(0.07)	1.46(0.09)	1.66(0.13))	2.50(0.70)	< 0.001[b,c,e,f,B,C,D,E,F,G,H,I,J]
**CSF Aβ42 (pg/mL)**	1642.4(400.0)	1781.9(663.7)	1748.7(497.6)	1987.4(829.5)	809.0(271.6)	811.3(191.3)	893.7(460.9)	731.9(396.8)	606.3(381.3)	< 0.001[I,α,β,γ,δ]
**CSF p-tau (pg/mL)**	20.6(8.31)	19.6(5.12)	20.3(6.24)	26.7(11.0)	19.0(4.85)	15.4(6.15)	22.4(11.6)	36.0(16.1)	37.2(17.8)	< 0.001[C,D,E,F,G,H,I]
**CSF t-tau (pg/mL)**	233.6(78.9)	221.3(53.5)	234.1(65.6)	294.5(94.8)	216.2(49.2)	172.9(66.5)	241.5(104.9)	353.2(133.4)	381.3(184.8)	< 0.001[C,D,E,F,G,H,I]
**Plasma NFL (pg/mL)**	27.4(9.31)	26.4(6.89)	35.9(14.5)	27.9(11.5)	40.9(15.5)	44.0(31.5)	39.8(23.5)	44.0(15.7)	51.6(19.5)	0.024

Continuous variables were expressed as mean (standard deviation) and categorical variables as number (%). P values of post hoc pairwise comparisons were only shown for groups in the same stage or in the same amyloid status.

Abbreviations: A+/-=abnormal or normal β-amyloid; CN = cognitively normal; EF = executive function; MCI = mild cognitive impairment; MMSE = mini-mental status examination; NFL = neurofilament light chain; t-tau = total tau; p-tau = phosphorylated tau.

Within A- groups:
a Stage0 vs Stage1 ; $P < 0.05$
b Stage0 vs Stage2 ; $P < 0.05$
c Stage0 vs Stage3 ; $P < 0.05$
d Stage1 vs Stage2 ; $P < 0.05$
e Stage1 vs Stage3 ; $P < 0.05$
f Stage2 vs Stage3 ; $P < 0.05$

Within A+ groups:
A Stage0 vs Stage1 ; $P < 0.05$
B Stage0 vs Stage2 ; $P < 0.05$
C Stage0 vs Stage3 ; $P < 0.05$
D Stage0 vs Stage4 ; $P < 0.05$
E Stage1 vs Stage2 ; $P < 0.05$
F Stage1 vs Stage3 ; $P < 0.05$
G Stage1 vs Stage4 ; $P < 0.05$
H Stage2 vs Stage3 ; $P < 0.05$
I Stage2 vs Stage4 ; $P < 0.05$
J Stage3 vs Stage4 ; $P < 0.05$

Within the same stage:
α A-Stage0 vs A+Stage0 ; $P < 0.05$
β A-Stage1 vs A+Stage1 ; $P < 0.05$
γ A-Stage2 vs A+Stage2 ; $P < 0.05$
δ A-Stage3 vs A+Stage3 ; $P < 0.05$

Supplementary Table 3. Numbers of participants included in linear mixed effects models

	Model 1	Model 2	
**Tau stage**	**All participants (n)**	**A- participants (n)**	**A+ participants (n)**
**MMSE**			
Stage 0	65	37	28
Stage 1	69	41	28
Stage 2	336	171	165
Stage 3	113	18	95
Stage 4	43	/	43
**Memory composite**			
Stage 0	62	34	28
Stage 1	67	40	27
Stage 2	319	164	155
Stage 3	106	15	91
Stage 4	39	/	39
**EF composite**			
Stage 0	62	34	28
Stage 1	67	40	27
Stage 2	320	163	157
Stage 3	104	15	89
Stage 4	39	/	39

Abbreviations: A+ = abnormal β-amyloid; A- = normal β-amyloid; EF = executive function; MMSE=mini-mental state examination

Supplementary Table 4. Comparisons of cognitive change between abnormal amyloid versus normal amyloid status

Tau Stage	Difference(95% CI)	P-value
**MMSE**		
Stage 0	-0.04(-0.64,0.56)	0.58
Stage 1	-0.004(-0.61,0.60)	0.92
Stage 2	-0.11(-0.34,0.13)	0.62
Stage 3	-0.22(-0.68,0.25)	0.59
**Memory composite**		
Stage 0	-0.05(-0.006,0.085)	0.2
Stage 1	-0.03(-0.08,0.01)	0.16
Stage 2	-0.02(-0.04,-0.003)	**0.02**
Stage 3	-0.008(-0.05,0.03)	0.67
**Executive function composite**		
Stage 0	-0.04(-0.06,0.05)	0.89
Stage 1	0.05(-0.45,0.56)	0.99
Stage 2	-0.03(-0.25,0.18)	0.38
Stage 3	-0.41(-0.96,0.14)	0.35

Analyses of cognitive change and comparisons in linear mixed effects models were adjusted for age, gender, education years and ApoE ε4 counts

Abbreviations: CI = confidence interval; MMSE = mini-mental state examination

Supplementary Table 5. Distributions of time points in longitudinal analyses of cognition

	MMSE					Memory composite					EF composite				
**Months**	**Stage 0**	**Stage 1**	**Stage 2**	**Stage 3**	**Stage 4**	**Stage 0**	**Stage 1**	**Stage 2**	**Stage 3**	**Stage 4**	**Stage 0**	**Stage 1**	**Stage 2**	**Stage 3**	**Stage 4**
**-144**	0	0	6	5	0	0	0	6	5	0	0	0	6	5	0
**-132**	3	1	15	10	0	3	1	15	10	0	3	1	15	10	0
**-120**	4	1	20	13	3	4	1	20	13	3	4	1	20	13	3
**-108**	4	1	26	15	3	4	1	26	15	3	4	1	26	15	3
**-96**	4	2	26	15	3	4	2	26	15	3	4	2	26	15	3
**-84**	5	3	42	19	4	5	3	42	19	5	5	3	42	19	5
**-72**	15	13	75	35	11	15	13	76	35	11	15	13	76	35	11
**-60**	18	18	100	41	15	18	18	100	41	15	18	18	100	41	15
**-48**	24	22	141	59	21	24	22	141	59	21	24	21	141	59	21
**-36**	16	20	113	45	20	16	20	113	45	20	16	20	113	44	20
**-24**	21	20	140	48	19	21	20	140	48	19	21	20	140	48	19
**-12**	7	7	61	25	13	7	7	61	25	13	7	7	61	24	13
**0**	65	69	336	113	43	62	67	319	106	39	62	67	320	104	39
**12**	25	18	158	70	28	14	5	75	32	10	13	5	73	27	10
**24**	21	20	120	42	9	3	4	40	15	4	3	4	39	15	4
**36**	5	2	34	10	2	0	0	16	1	0	0	0	16	1	0

Minus numbers in 'Month' column means the time points before the baseline.

Abbreviations: EF = executive function; MMSE=mini-mental state examination

Supplementary Figure 1. Flow chart of study participants.

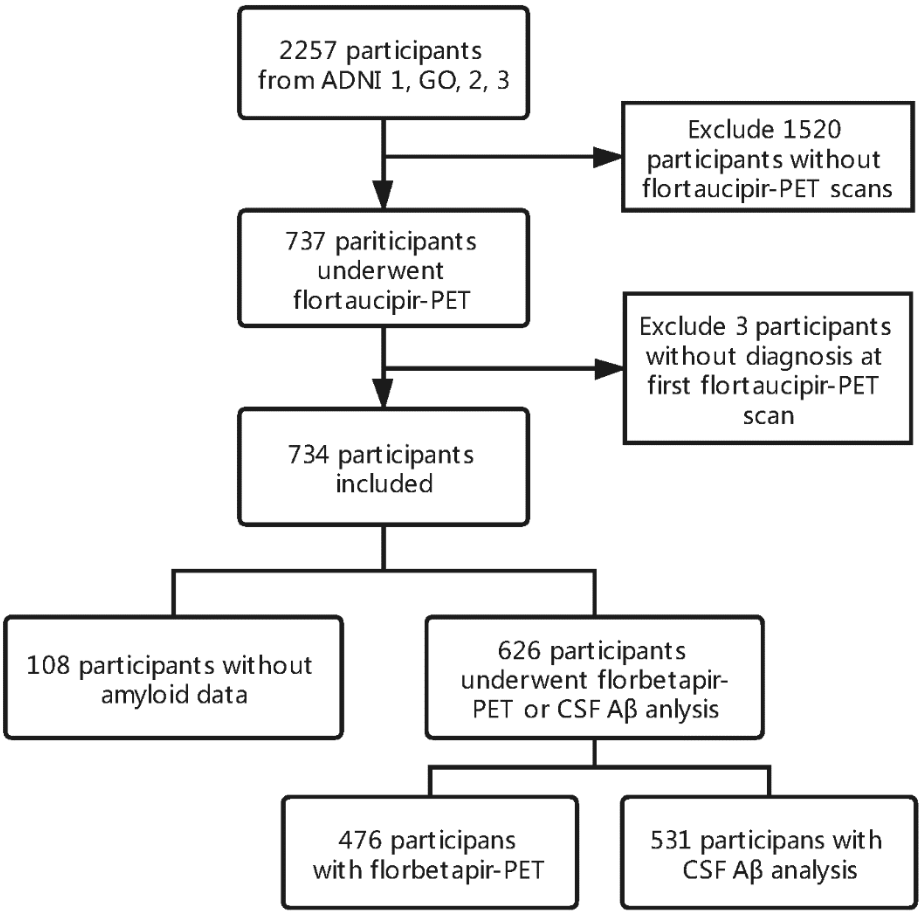

Abbreviation: ADNI = Alzheimer’s Disease Neuroimaging Initiative; Aβ = β-amyloid.

Supplementary Figure 2. Braak ROIs used for tau staging.

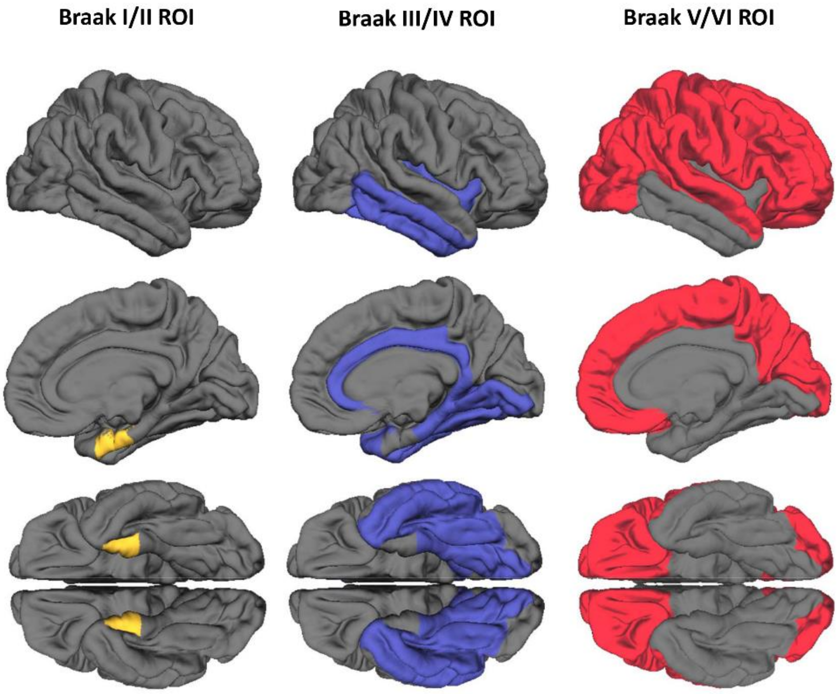

Braak ROI I/II, Braak ROI III/IV, and Braak ROI V/VI were labeled with different colors from lateral, medial, and inferior views of cerebrum. From ROI V/VI to Braak ROI I/II, composite SUVrs of flortaucipir were successively used to assign stages. A composite SUVr > 1.873 for Braak V/VI ROI was used to assign stage 4; > 1.523 for Braak III/IV ROI to assign stage 3; > 1.304 for Braak III/IV ROI to assign stage 2; > 1.129 for Braak I/II ROI to assign stage 1; ≤1.129 for Braak I/II ROI to assign stage 0 (staging results shown in Supplementary Fig.3).

Abbreviation: ROI = region of interest; SUVr = standard uptake value ratio.

Supplementary Figure 3. Flow chart of in vivo staging using flotaucipir PET.

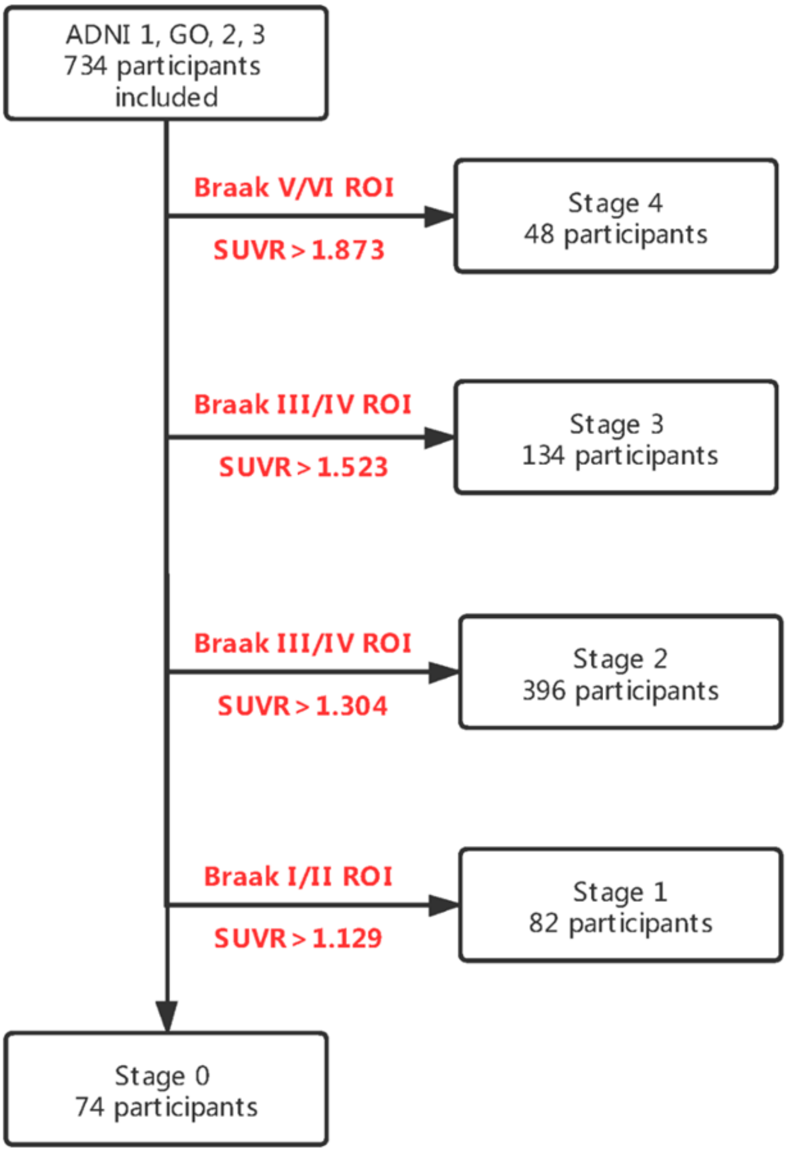

Abbreviation: ADNI = Alzheimer's Disease Neuroimaging Initiative; SUVR = standard uptake value ration; ROI = region of interest
